# Acknowledgement to Reviewers of *Healthcare* in 2014

**DOI:** 10.3390/healthcare3010001

**Published:** 2015-01-08

**Authors:** Healthcare Editorial Office

**Affiliations:** MDPI AG, Klybeckstrasse 64, CH-4057 Basel, Switzerland

The editors of *Healthcare* would like to express their sincere gratitude to the following reviewers for assessing manuscripts in 2014:

Adams, James B.
Ádány, R.
Anthony, Denis
Appleton, Katherine
Baba, E
Bandyopadhyay, Mridula
Barnard, Neal
Bavari, Sina
Beerthuizen, Annemerle
Belasco, Eric
Berry, Diane C
Berry, Robert J
Biasi, Fiorella
Blumenthal, David
Borkow, Gadi
Bowen, Judith
Bravaccio, Carmela
Bristow, Ivan R
Bruning, Harry
Burdick, Anne
Büscher, Christian
Cantiello, John
Cárdenas, Washington B.
Chao, Yann-Fen C.
Chin, K Y
Choo, Tze Siang
Crotty, Bradley H.
Dattakumar, Ambica
Demarré, L.
Dougherty, Denise
Dubuy, Veerle
Dunbar, James D
Dworkin, Anthony Gary
Edwards, Helen
Elbadawy, Hossein
Flechtner-Mors, Marion
Francisco, Pedro García-Fernández
Frye, Richard E.
Funnell, Martha M.
Garfield, Monica J.
Gethin, Georgina
Ghernaout, Djamel
Golemati, Spyreta
Gunningberg, Lena
Haan, Mary N.
Haglind, E.
Hammer, T. R.
Harrington, Daniel W
Heinen, Maud
Hennigar, Steve
Herendeen, Neil
Holick, Michael F
Horton, Mark
Hosking, Diane
Houghton, Pamela E.
House, Kim.
Howard, M. A.
Idzenga, Tim
Isermann, Berend
Jaul, Ephraim
John, Bruce
Jørgensen, Marie Birk
Junaid, Mohammed A.
Juonala, Markus
Kawasaki, Lisa
Khalil, Hanan
Kim, Woo-Yang
Knechtle, Philipp
Koel, Gerard
Kretschmer, Lutz
Kuhn, Jens
Lai, Julian C L
Lanigan, Julie
Leroy, Gondy
Levinson, Howard
Lo, Shu-Fen
Lohela-Karlsson, Malin
Lymberopoulos, Dimitrios K.
Manzano, F.
Mark, Katrina S.
Martinez, Homero
Mccaughan, Dorothy
McCloughen, Andrea
Mcclung, James
McGrady, Meghan E.
McManus, Alexandra
Menzies, Victoria
Millar, Lynne
Miller, Carol T.
Moore Jr., Arl Van
Murdaca, Giuseppe
Nygard, Clas-Håkan
Ousey, Karen
Pantanowitz, Liron
Pennathur, Priyadarshini R.
Peters, Christian Daugaard
Pu, Lee Q
Quinn, Edel Marie
Rahman, Mushtaq
Ramos, Victoria
Robitaille, Julie
Rossignol, Dan
Ruggiero, Ken
Russell, Maria
Ryu, Seewon
Schelble, Jenni L.
Schultz, Meleesa J.
Schulze, Kerry
Shand, Fiona
Shore, Jay
Silva, Edibaldo
Sint, Tin Tin
Smerdon, Gary
Sogaard, Erik
Sprigle, Stephen
Stanley, Susanne H.
Steinhardt, Mary A.
Tannen, Antje
Thompson, Juliana
Thraen, Iona
Tsai, Jing-Jane
Ubbink, Dirk T.
Ueno, Takehisa
Walsh, William R.
Welsh, Jean
Whitty, Jennifer A.
Xu, Fujie
Yan, Qing
Yen, Chia-Hung
Yu, Ping
Zlokovic, Berislav

